# An analysis of adjuvant chemoradiotherapy versus chemotherapy on the survival rates for patients with stage IB-III uterine serous carcinoma

**DOI:** 10.1038/s41598-024-53172-3

**Published:** 2024-03-11

**Authors:** Shuqing Li, Zhihui Yi, Mingqing Li, Zhiling Zhu

**Affiliations:** Department of Obstetrics and Gynecology, Obstetrics and Gynecology Hospital of Fudan University, 128 Shenyang Road, Shanghai, 200090 China

**Keywords:** Cancer, Medical research, Oncology

## Abstract

The aim of the present study was to investigate whether a combination of chemotherapy plus radiotherapy was able to increase the overall survival rates compared with chemotherapy alone in stage IB-III uterine serous carcinoma. A total of 1096 patients (593 who had not received radiotherapy, and 503 who had) with primary stage IB-III uterine serous carcinoma who underwent surgery and received chemotherapy were included in the present study. The Kaplan–Meier method and Log-Rank tests showed that radiotherapy did not increase 5-year overall survival rates compared with the no-radiotherapy groups (52.3 cf. 50.8%, respectively; *P* = 0.641). Cox regression analysis subsequently corroborated that radiotherapy did not affect the 5-year overall survival rate (*P* = 0.635). Patients who were aged ≥ 60 years had a higher mortality rate [hazard ratio (HR), 1.712; 95% confidence interval (95% CI), 1.385–2.117; *P* < 0.05]. The 5-year overall survival rates were found to be lower in the groups where the regional lymph nodes had not been removed (HR 0.645; 95% CI 0.508–0.821; *P* < 0.05). Chemotherapy plus radiotherapy was found to not be associated with improved 5-year overall survival rates. However, chemotherapy may be a better treatment option for patients with primary stage IB-III uterine serous carcinoma who have undergone surgery.

## Introduction

Uterine endometrioid carcinoma is the most common histological type of endometrial carcinoma, which tends to have a favorable prognosis, typically presenting at an early stage with abnormal uterine bleeding^1,2^. Uterine serous carcinoma only accounts for a very small percentage of cases of endometrial carcinomas compared with endometrioid carcinoma^3^. Abnormal uterine bleeding is the most common clinical presentation for uterine serous carcinoma. Patients with uterine serous carcinoma have different characteristics compared with those with endometrioid carcinoma. They tend to be older at the time of diagnosis, nonobese and parous^4,5^. Uterine serous carcinoma has a higher propensity for lymphovascular invasion, and intraperitoneal as well as extra-abdominal spread, compared with endometrioid carcinoma^6^. Uterine serous carcinoma tends to present at an advanced stage. Approximately 70% of patients with uterine serous carcinoma present with either stage III or stage IV disease^7^. Uterine serous carcinoma is surgically staged in the same manner as endometrioid carcinoma, and surgical staging is performed for uterine serous carcinoma due to the high risk of nodal and extrauterine metastasis. Optimal cytoreduction is an important component of the surgical treatment method^8^.

For patients who undergo surgery, the adjuvant treatment approach is usually stratified, based on the risk of disease recurrence, which is determined by taking into account the stage of disease, histology of the tumor, and other pathological factors. Patients with serous carcinoma are deemed to be at high risk, regardless of the disease stage^9^. The presence of lymphovascular space invasion (LVSI) is an independent risk factor for lymph node metastasis and disease recurrence. Additionally, having an older age has been associated with higher rates of clinical failure and worse survival. Patients with serous carcinoma undergoing surgery have a relatively poor prognosis following hysterectomy alone. Even among patients with completely surgically staged, node-negative, stage I uterine serous carcinoma, the 5-year overall survival rate is only ~ 70%^10,11^. Therefore, adjuvant treatment is often administered even though the optimal therapy following surgery with respect to overall survival in the majority of situations remains unclear^12^. In view of this, weighing the survival benefits of adjuvant treatment against the increased risk of adverse events remains an important consideration. Severe adverse events (such as nausea, vomiting, diarrhea, tingling, numbness, fatigue and hair loss) may affect the quality of the patients’ life following adjuvant treatment^13^.

Various types of adjuvant therapies have been proposed for patients following surgical treatment for uterine serous carcinoma; however, available data specifically for patients with uterine serous carcinoma are limited due to the relative rarity of this histological subtype. Studying the effect of chemotherapy in uterine serous carcinoma is important due to the high risk of metastasis, recurrence and poor prognosis^14,15^. Improvements in survival outcome with chemotherapy have been documented in certain studies^16–18^. The aim of the present paper was to evaluate the effect of chemotherapy plus radiotherapy in patients with stage IB-III uterine serous carcinoma compared with chemotherapy alone.

## Methods

**Figure 1:**
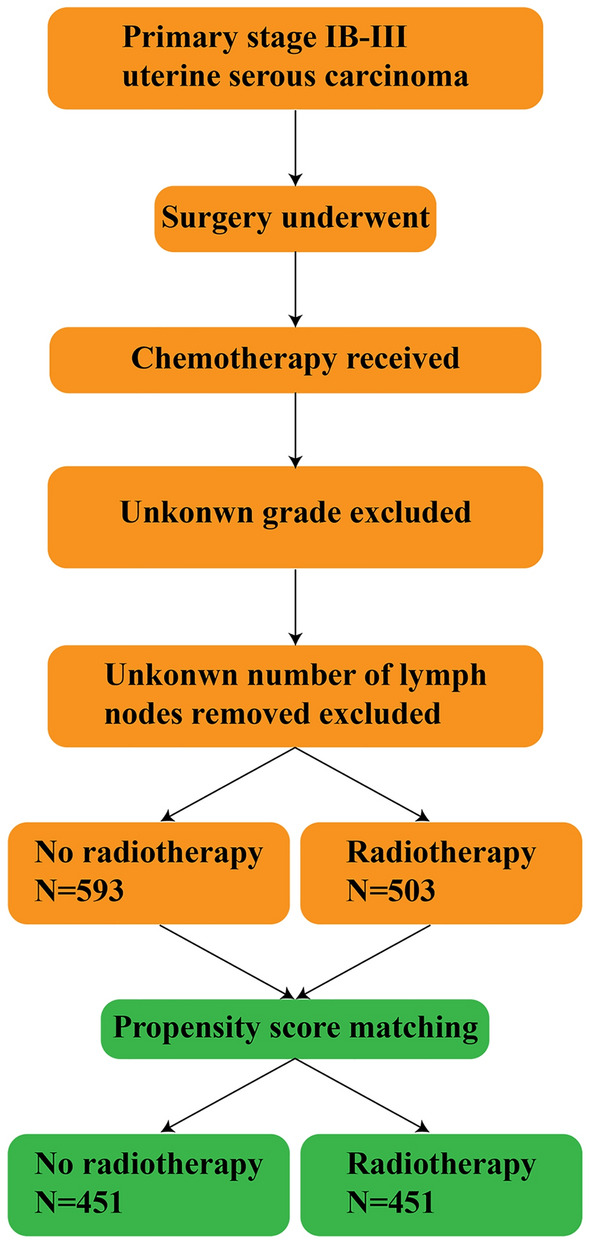
Flow chart.

Data were extracted from the Surveillance, Epidemiology and End Results (SEER) database between 2004 and 2015. Patients with primary stage IB-III uterine serous carcinoma were identified. All the selected subjects underwent surgery and received chemotherapy. The numbers of lymph nodes removed from the patients were available. The diagnosis of the condition depended on cytological or histological analysis. A total of 1,096 patients with stage IB-III uterine serous carcinoma were included in the present study. The following variables were included: Radiotherapy, age, stage of carcinoma, grade, tumor size, regional lymph nodes removed (or not), and the survival months. In the extracted dataset, radiotherapy was categorized as ‘yes’ or ‘no’. The patents’ ages were divided into two groups: < 60 and ≥ 60 years. The stage of carcinoma was classified into stages IB, II or III, and the grades were grouped into grade I/II or grade III/IV. Tumor sizes were categorized into three groups: < 2 cm, ≥ 2 cm and unknown size. Regional lymph nodes removed (or not) were categorized as ‘none’ and ‘removed’ groups. Finally, the subjects were assessed for the association between radiotherapy and 5-year overall survival rates (Fig. 1).

SPSS software version 26 (IBM Corp.) was used to perform the statistical analysis. The univariate associations between categorical variables and radiotherapy were assessed using Pearson’s χ2-tests. All statistical tests were two-sided tests. *P* < 0.05 was considered to indicate a statistically significant difference. Propensity score matching (PSM) was used to minimize selection bias, which is both common and inevitable in retrospective studies. The categorical variables were as follows: Age, stage, grade, tumor size, and regional lymph nodes removed (or not), and differences between the radiotherapy and no-radiotherapy groups were matched using 1:1 match ratio method. Survival analysis was performed using the Kaplan–Meier method and log-rank tests. Finally, the Cox proportional hazards model was used to estimate the hazard ratio (HR) and 95% confidence intervals (95% CI).

### Consent for publication

Yes.

## Results

### Patient characteristics

A total of 1,096 patients (593 who had not received radiotherapy, and 503 who had) with primary stage IB-III uterine serous carcinoma met the study inclusion criteria. Among these patients, 21.2% were aged < 60 years, and 78.8% were aged ≥ 60 years. A total of 26.0% of the patients were stage IB, 12.0% were stage II, and 62.0% were stage III. In addition, 26.7% of the patients were grade I/II, and 73.3% were grade III/IV. A total of 10.9% of the patients had tumors measuring < 2 cm in size, whereas 54.2% of them had tumors measuring ≥ 2 cm. Regional lymph nodes were removed from 69.9% of the patients. Nearly half (48.7%) of the patients had an overall survival rate of 5 years (Table 1).

**Table 1:** Demographics for patients with stage IB-III uterine serous carcinoma.

Characteristics	No	%
Radiotherapy		
No	593	54.1
Yes	503	45.9
Age		
< 60 y	232	21.2
≥ 60 y	864	78.8
Stage		
IB	285	26.0
II	132	12.0
III	679	62.0
Grade		
I/II	293	26.7
III/IV	803	73.3
Tumor size		
< 2 cm	120	10.9
≥ 2 cm	594	54.2
Unknown	382	34.9
Lymph node		
None	330	30.1
Removed	766	69.9
Survival months		
< 60 m	562	51.3
≥ 60 m	534	48.7

Laterality: the side of ovaries on which the primary tumor originated.

### Univariate categorical variables

Patients with primary stage IB-III uterine serous carcinoma in the radiotherapy groups tended to be younger compared with those in the no-radiotherapy groups (for patients aged < 60 years, 21.9 cf. 20.6%, respectively; and for patients aged ≥ 60 years, 78.1 cf. 79.4%, respectively; *P* = 0.601). Fewer patients were found at stage III in the radiotherapy groups compared with those in the no-radiotherapy groups (for all the included stages: Stage IB, 27.6 cf. 24.6%, respectively; stage II, 14.5 cf. 9.9%, respectively; and stage III, 57.9 cf. 65.4%, respectively; *P* < 0.05). A higher number of patients were grade I/II in the radiotherapy groups compared with those in the no-radiotherapy groups (grade I/II, 28.2 cf. 25.5%, respectively; for grade III/IV, 71.8 cf. 74.5%, respectively; *P* = 0.302). Fewer patients had tumors measuring < 2 cm, whereas more patients had tumors measuring ≥ 2 cm, in the radiotherapy groups compared with the no-radiotherapy groups (< 2 cm, 8.2 cf. 13.3%, respectively; and ≥ 2 cm, 55.3 cf. 53.3%, respectively; *P* < 0.05). Finally, there were more patients with regional lymph nodes removed in the radiotherapy groups compared with the no-radiotherapy groups (79.5 cf. 61.7%, respectively; *P* < 0.05). Selection bias and non-uniformity between the radiotherapy and no-radiotherapy groups were eliminated using PSM. Statistical analysis was further performed for the equalized number of patients (n = 451). The categorical variables were found to be well balanced, and the potential covariates between the two groups were markedly decreased (Table 2).

**Table 2:** Comparison of univariate covariates for patients with stage IB-III uterine serous carcinoma.

Characteristics	Before PSM			After PSM		
	Radiotherapy-(n = 593)	Radiotherapy + (n = 503)	*P*	Radiotherapy-(n = 451)	Radiotherapy + (n = 451)	*P*
Age			0.601			0.295
< 60 y	122 (20.6)	110 (21.9)		92 (20.4)	105 (23.3)	
≥ 60 y	471 (79.4)	393 (78.1)		359 (79.6)	346 (76.7)	
Stage			0.017			0.235
IB	146 (24.6)	139 (27.6)		127 (28.2)	112 (24.8)	
II	59 (9.9)	73 (14.5)		50 (11.1)	65 (14.4)	
III	388 (65.4)	291 (57.9)		274 (60.8)	274 (60.8)	
Grade			0.302			0.541
I/II	151 (25.5)	142 (28.2)		119 (26.4)	111 (24.6)	
III/IV	442 (74.5)	361 (71.8)		332 (73.6)	340 (75.4)	
Tumor size			0.022			0.057
< 2 cm	79 (13.3)	41 (8.2)		51 (11.3)	39 (8.6)	
≥ 2 cm	316 (53.3)	278 (55.3)		229 (50.8)	264 (58.5)	
Unknown	198 (33.4)	184 (36.6)		171 (37.9)	148 (32.8)	
Lymph node			< 0.01			0.753
None	227 (38.3)	103 (20.5)		107 (23.7)	103 (22.8)	
Removed	366 (61.7)	400 (79.5)		344 (76.3)	348 (77.2)	

PSM: propensity score matching. *P *value < 0.05 is regarded as statistically significant.

### Kaplan–Meier analysis

The association between univariate categorical variables and 5-year overall survival rates was analyzed using the Kaplan–Meier method and log-rank tests. For patients with primary stage IB-III uterine serous carcinoma, administering radiotherapy did not significantly increase the 5-year overall survival rates compared with the no-radiotherapy group (52.3 cf. 50.8%, respectively; *P* = 0.641). There were, however, significant differences in 5-year overall survival rates when comparing between the < 60 years and ≥ 60 years groups (59.9 cf. 49.2%, respectively; *P* < 0.05). The stage of the carcinoma was found to be a risk factor for the 5-year overall survival rates (stage IB, 68.2%; stage II, 62.6%; and stage III, 42.0%; *P* < 0.05). No significant differences in 5-year overall survival rates were found between grade I/II and grade III/IV carcinomas (47.8 cf. 52.8%, respectively; *P* = 0.190). Having tumors < 2 cm in size was associated with a higher 5-year overall survival rate compared with the group with tumors ≥ 2 cm (66.7 cf. 49.1%, respectively; *P* < 0.05). Finally, having the regional lymph nodes removed led to an improvement in the 5-year overall survival rates (*P* < 0.05) (Table 3).

**Table 3:** Univariate analysis of clinical factors with 5-year overall survival for patients with stage IB-III uterine serous carcinoma.

Characteristics	No	5-year OS (%)	*P*
Radiotherapy			0.641
No	229	50.8	
Yes	236	52.3	
Age			< 0.01
< 60 y	118	59.9	
≥ 60 y	347	49.2	
Stage			< 0.01
IB	163	68.2	
II	72	62.6	
III	230	42.0	
Grade			0.190
I/II	110	47.8	
III/IV	355	52.8	
Tumor size			< 0.01
< 2 cm	60	66.7	
≥ 2 cm	242	49.1	
Unknown	163	51.1	
Lymph node			< 0.01
None	83	39.5	
Removed	382	55.2	

### Cox regression analysis

The correlation between the categorical variables and 5-year overall survival was further investigated using univariate Cox regression analysis. For patients with primary stage IB-III uterine serous carcinoma, administering radiotherapy did not increase the 5-year overall survival rate compared with the no-radiotherapy groups (*P* = 0.663). However, having an age ≥ 60 years did lead to a significant reduction in the 5-year overall survival rate (HR 1.752; 95% CI 1.418–2.166; *P* < 0.05). Regional lymph nodes unremoved were associated with a lower 5-year overall survival rate (HR 0.615; 95% CI 0.484–0.782; *P* < 0.05). Finally, stage, grade and tumor size were found to exert no influence on the 5-year overall survival rate (*P* = 0.976, *P* = 0.591 and *P* = 0.530, respectively) (Table 4).

**Table 4:** Univariate cox regression analysis for 5-year overall survival for patients with stage IB-III uterine serous carcinoma.

Characteristics	HR (95% CI)	*P*
Radiotherapy		0.663
No	Ref	
Yes	–	
Age		< 0.01
< 60 y	Ref	
≥ 60 y	1.752 (1.418–2.166)	
Stage		0.976
IB	Ref	
II	–	
III	–	
Grade		0.591
I/II	Ref	
III/IV	–	
Tumor size		0.530
< 2 cm	Ref	
≥ 2 cm	–	
Unknown	–	
Lymph node		< 0.01
None	Ref	
Removed	0.615 (0.484–0.782)	

HR: hazard ratios; CI: confidence intervals; Ref: reference. *P *value < 0.05 is regarded as statistically significant.

To investigate how the categorical variables jointly affected 5-year overall survival, these factors were incorporated into the multivariate Cox regression analysis. For patients with primary stage IB-III uterine serous carcinoma, administering radiotherapy continued to have no effect on the 5-year overall survival rates (*P* = 0.635). Having an age ≥ 60 years, however, did result in higher mortality rates (HR 1.712; 95% CI 1.385–2.117; *P* < 0.05). Finally, the 5-year overall survival rates were lower in the regional lymph nodes-unremoved group (HR 0.645; 95% CI 0.508–0.821; *P* < 0.05) (Table 5).

**Table 5:** Multivariate cox regression analysis for 5-year overall survival for patients with stage IB-III uterine serous carcinoma.

Characteristics	HR (95% CI)	*P*
Radiotherapy		0.635
No	Ref	
Yes	–	
Age		< 0.01
< 60 y	Ref	
≥ 60 y	1.712 (1.385–2.117)	
Lymph node		< 0.01
None	Ref	
Removed	0.645 (0.508–0.821)	

HR: hazard ratios; CI: confidence intervals; Ref: reference. *P *value < 0.05 is regarded as statistically significant.

## Discussion

The most appropriate adjuvant treatment of surgically resected uterine serous carcinoma at present remains controversial. There is a lack of high-level evidence in terms of managing these patients. The present study was limited to patients with stage IB-III uterine serous carcinoma who underwent surgery and received chemotherapy. The findings of the study have revealed that radiotherapy is not associated with improved 5-year overall survival rates for patients with stage IB-III uterine serous carcinoma.

Retrospective studies have reported improvements in overall survival and disease-free survival rates with chemotherapy for stage I-IV uterine serous carcinoma^17,18^. Donkers et al. observed benefits of chemotherapy in all stages of uterine serous carcinoma. Patients with uterine serous carcinoma who received chemotherapy showed improved survival rates compared with those who received radiotherapy, chemoradiotherapy or no adjuvant treatment (a total of 272 patients were included in their study)^19^. Subsequently, Matei et al. reported that, with the enrollment of 131 patients with uterine serous carcinoma, chemoradiotherapy was found not to be associated with longer survival rates compared with chemotherapy alone in patients with stage III or IVA endometrial carcinoma^20^. Furthermore, Susumu et al. found that administering chemotherapy led to improved progression-free survival and overall survival rates for patients with intermediate-risk or high-risk endometrial carcinoma compared with radiotherapy^21^. By contrast, Kurnit et al. held the opinion that brachytherapy with or without chemotherapy was associated with improved survival, and should therefore be considered for patients with stage IA-II uterine serous carcinoma following surgery. However, additionally, this study observed that chemotherapy alone was also associated with an improvement in the overall survival rate compared with no adjuvant treatment (a total of 737 patients were included)^3^. In the subgroup analysis of Chodavadia et al., chemoradiotherapy was found to be associated with improved overall survival rates compared with radiotherapy alone for patients with high-intermediate and high-risk stage IB endometrial carcinomas. However, no overall survival benefits were identified for stages IA or II endometrial carcinomas (enrolling 852 patients with stage IA–II uterine serous carcinoma)^22^. The results of a study by de Boer et al., in which 105 patients with stage IA–III uterine serous carcinoma were enrolled, showed that a significant improvement in overall survival was observed with chemoradiotherapy compared with radiotherapy alone. However, one limitation of that study was an acknowledgement that their results were not powerful, since the number of patients was too small to have enabled reporting on treatment efficacy across the different stages of uterine serous carcinoma^23^. The inconsistency between adjuvant treatment and survival outcome was possibly associated with the number of patients with uterine serous carcinoma in these subgroup analyses being relatively small, and uterine serous carcinoma has often been grouped together with other histological types. It is noteworthy that none of these studies were as large as the present study. In addition, another factor to consider is that different chemotherapy and radiotherapy regimens were used among the different trials.

The present study has shown that, for patients with primary stage IB-III uterine serous carcinoma who underwent surgery and received chemotherapy, no significant differences in 5-year overall survival rates were observed between the radiotherapy and no-radiotherapy groups (*P* = 0.641). The 5-year overall survival rates were significantly (*P* < 0.05) reduced for patients in the age ≥ 60 years, stage II/III carcinoma, tumors ≥ 2 cm and regional lymph nodes-unremoved groups, which was consistent with the findings of the majority of previously published studies. Cox regression analysis showed that radiotherapy continued to exert no significant effect on the 5-year overall survival rates (*P* = 0.635) after having excluded confounding factors. The patients’ age and their status of having the regional lymph nodes removed or not were significant for overall survival, whereas stage and grade of carcinoma and tumor size were not found to be significant prognostic factors for overall survival.

Importantly, Kaplan–Meier and log-rank analyses demonstrated that stage II/III carcinoma and tumors ≥ 2 cm were associated with a decrease in the 5-year overall survival rates. Stage of carcinoma and tumor size separately affected the distribution of 5-year overall survival rates; however, Cox regression analysis indicated that the stage and tumor size exerted no influence on 5-year overall survival. Kaplan–Meier and log-rank analyses were used to investigate the association between univariate categorical variables and 5-year overall survival rates, not including the impact of other categorical variables. The Cox regression model extends survival analysis to simultaneously assess the effects of several variables on the 5-year overall survival rate. Our conclusions were reached for patients under the condition that they had stage IB-III uterine serous carcinoma, underwent surgery and received chemotherapy. Our findings that stage of carcinoma and tumor size were risk factors for survival were found not to be contradictory with those of previous studies.

The strengths of the present study were that it included a large number of patients, and it has provided authoritative evidence on the efficacy of radiotherapy for treating patients with stage IB-III uterine serous carcinoma who underwent surgery and received chemotherapy. Additionally, all subjects were randomly enrolled without subjective preference, and PSM was used to eliminate possible inherent bias, which resulted in objective conclusions.

However, there were also several limitations associated with the present study. First, the SEER database lacked detailed information regarding surgery. Furthermore, the presence (or not) of LVSI was unknown. Despite these limitations, the present study remains, to the best of the authors’ knowledge, one of the largest and most specialized retrospective studies on uterine serous carcinoma, and has contributed to the burgeoning body of literature that serves to guide adjuvant treatment decisions.

An improved selection of therapeutic options for adjuvant treatment could be achieved by the integration of molecular characteristics. Uterine serous carcinomas are characterized by a high frequency of TP53 mutations. These alterations occur relatively early during tumor progression, and may serve as molecular markers for the early detection of uterine serous carcinoma^24^. Furthermore, human epidermal growth factor receptor 2 (HER2) is a prognostic biomarker and therapeutic target in uterine serous carcinoma. Approximately one-third of all uterine serous carcinomas have been shown to overexpress HER2 protein. Survival benefits from utilizing a combination of chemotherapy and anti-HER2 targeted therapy have been reported for patients with advanced or recurrent uterine serous carcinoma^25^.

In conclusion, given our results and the known toxicity of radiotherapy, chemotherapy alone should be considered as an option for patients with stage IB-III uterine serous carcinoma who underwent surgery. However, further confirmation of the benefits of chemotherapy, preferably in a prospective trial as well as in the guidance of molecular characteristics, is warranted.

## Data Availability

The datasets analyzed during the present study are available from the corresponding author on reasonable request.
